# Hepatic subcapsular hematoma: A rare complication post-ERCP; a case report

**DOI:** 10.1097/MD.0000000000037705

**Published:** 2024-03-29

**Authors:** Georgio El Koubayati, Tatiana Charbel, Antoine Aoun, Randa Choueiry

**Affiliations:** a Faculty of Medical Sciences, Lebanese University, Hadath Campus, Beirut, Lebanon; b Faculty of Medical Sciences, Université Saint Joseph, Beirut, Lebanon; c Gastroenterology Department, Dr. Serhal Hospital, Beirut, Lebanon.

**Keywords:** ERCP, ERCP complication, hepatic hematoma

## Abstract

**Introduction:**

Endoscopic retrograde cholangiopancreatography (ERCP) is commonly used in gastroenterology wards for both diagnostic and therapeutic purposes. It doesn’t however come free of complications. As a matter of fact, complications are reported in up to 10% of patients undergoing ERCP.

**Patient Concerns:**

In this article, we report the case of a patient who underwent ERCP and sphincterotomy for choledocholithiasis. Twenty-four hours after the procedure, the patient developed sudden sharp abdominal pain and dropped her hemoglobin levels.

**Diagnosis:**

An emergent gastroscopy was done and it ruled out bleeding from the sphincterotomy. Computed tomography of the abdomen showed a large hepatic subcapsular hematoma.

**Interventions:**

Blood was urgently transfused and the patient was transferred to the intensive care unit for monitoring.

**Outcomes:**

The patient’s condition quickly deteriorated despite extensive resuscitative measures, and eventually passed away on day 4 post ERCP.

**Lessons:**

Hepatic subcapsular hematoma is a very rare but fatal complication after ERCP and should be ruled out in patients who underwent the procedure and develop sudden abdominal pain with hemodynamic and laboratory instability.

## 1. Introduction

Endoscopic retrograde cholangiopancreatography is a minimally invasive procedure used in the management of biliary and pancreatic diseases. It is considered less invasive and more cost-effective than surgical alternatives. It has nevertheless the highest rate of complications among the other endoscopic procedures, such as acute pancreatitis, post sphincterotomy bleeding, duodenal perforation, infection (cholangitis),^[1]^ and last but not least, hepatic subcapsular hematoma (HSH). The risks and complications associated with endoscopic retrograde cholangiopancreatography (ERCP) have caused its primary role to be switched from diagnostic to therapeutic.^[2]^ According to a recent multicentric database registry for ERCP in Japan, the overall complication rate of ERCP is 10.6%, with a 30-day mortality rate of 1.1%.^[3,4]^ Post-ERCP HSH is an extremely rare complication that was first described in the year 2000^[5]^ and has only a few case reports in the literature.^[6]^

We present the case of a 79-year-old female patient who developed a 37 mm HSH 24 hours after ERCP.

## 2. Case report

Seventy-nine-year-old woman presented to the emergency department of a Lebanese hospital for a 4 hours history of right upper quadrant pain accompanied with nausea and several episodes of nonbilious vomiting. She was febrile upon admission (38.2°C), with a blood pressure of 128/64, heart rate of 98 bpm, saturation of 97% breathing room air. Her medical history includes diabetes mellitus, hypertension, and iron deficiency anemia. She doesn’t smoke and doesn’t drink alcohol. She has no known food or drug allergies. Her physical examination was normal except for right upper quadrant tenderness and a positive Murphy sign. Urgent bedside echography showed a thickened gallbladder wall with multiple intravesicular stones and a dilated (around 10 mm) common bile duct.

**Figure 1.**
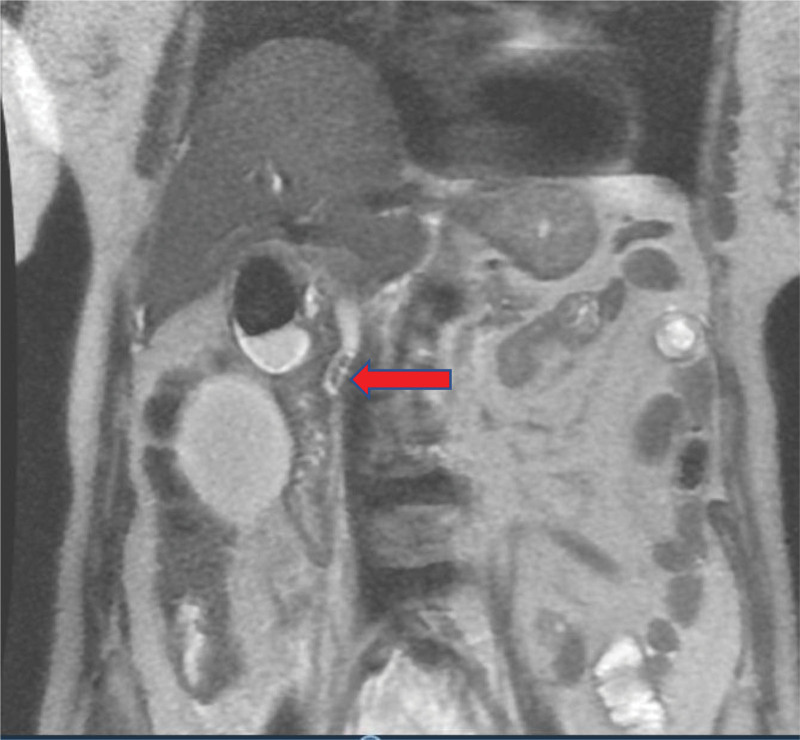
Coronal magnetic resonance imaging (MRI) showing a dilated (12 mm) common bile duct with a multiple filling defects in the proximal common bile duct (red arrow) indicating lodged stones.

Blood was withdrawn and showed the following results: hemoglobin 10.4 g/dL (reference range = 13–14 g/dL), mean corpuscular volume 81 fl (80–94 fl), white blood cell count 15,100 (4800–10,800) with 91% neutrophilia, platelet count 272 × (150–450 ×). Her creatinine level was 0.9 mg/dL. Her liver function tests showed: total bilirubin 1.4 mg/dL (0–1.3 mg/dL), with a direct bilirubinemia of 1.0 mg/dL (0–0.3 mg/dL), alkaline phosphatase 110 u/L (32–92 u/L), gamma GT 91 (7–64 u/L), alanine aminotransferase 12 u/L (6–40 u/L), aspartate aminotransferase 13 u/L (10–42 u/L), international normalized ratio 1.08, albumin 3.3 g/dL (3.5–5.0 g/dL). Her C-reactive protein was 58 mg/L (0–5 mg/L). She was started on intravenous hydration and analgesia as needed (acetaminophen 1g IV q8h PRN) and ceftriaxone 2g IV once daily and metronidazole 500 mg IV 3 times daily. She underwent a magnetic resonance cholangiopancreatography which showed a dilated common bile duct (12 mm) with multiple intraductal stones including a large stone estimated at 10 mm lodged in the proximal common bile duct (Fig. 1). The next day she underwent a therapeutic ERCP with sphincterotomy, then was transferred back to the surgical ward.

**Figure 2.**
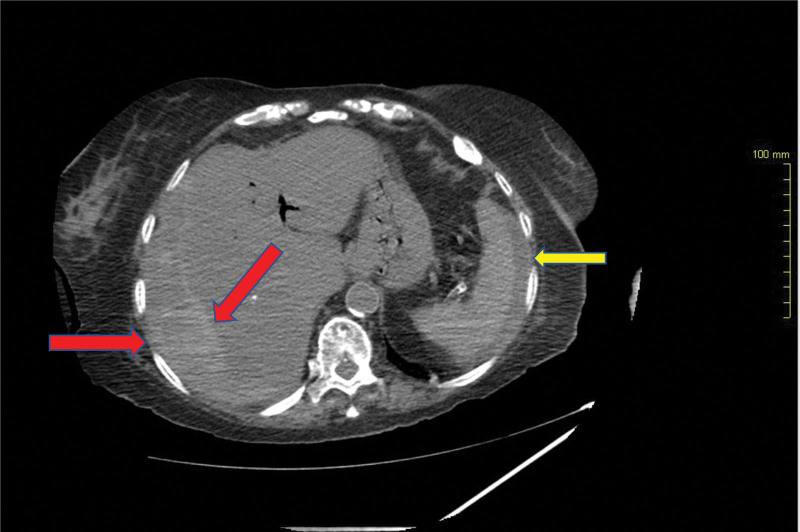
Axial view of computed tomography (CT) scan showing a subcapsular hepatic hematoma involving the right hepatic lobe (red arrows) measuring around 37 mm in maximum thickness with perisplenic fluids (yellow arrow) indicating intra-abdominal ascites.

The next day she became hypotensive (blood pressure = 80/40), tachycardic (heart rate = 120 bpm), and pale, and complained of severe sharp right upper quadrant pain. Her abdomen didn’t show signs of rigidity or guarding. An urgent complete blood count showed a hemoglobin of 6.4. She had no hematemesis nor melena; a digital rectal exam was negative for blood and fecal immunochemical test didn’t show hemoglobin. She was urgently transfused 2 units of isogroup red blood cells. An urgent upper gastrointestinal endoscopy ruled out bleeding from the sphincterotomy and a CT scan of the abdomen showed an HSH of 37 mm thickness involving the right hepatic lobe (Fig. 2).

Due to decreased level of consciousness and inability to protect her airways, the patient was intubated, sedated, and put on mechanical ventilation. Her hypovolemic shock kept worsening and led to acute kidney failure on day 3 post-ERCP, despite multiple blood transfusions, fluid infusions, and starting vasopressors. Her creatinine levels quickly climbed to 3.5 mg/dL leading to anuria, with hyperkalemia refractory to chelators, fluids, bicarbonate infusions, and furosemide. Her liver function also deteriorated with alanine aminotransferase and aspartate aminotransferase reaching 1073 and 1833 u/L, respectively, and international normalized ratio elevated to 3.5, resistant to vitamin K and fresh frozen plasma transfusions. Surgical interventions were deemed as very risky. Urgent dialysis was initiated. However, the patient kept deteriorating and passed away on day 4 post ERCP.

## 3. Discussion

Only 2% of ERCPs and sphincterotomies are complicated by major and clinically significant bleeding.^[6]^ However, an HSH is a very rare occurrence that was first described in 2000 by Ortega et al, with subsequent case reports reporting a fatality rate as high as 7.5%.^[5,7]^ The incidence of this complication is probably underestimated as most patients undergo ERCP without subsequent symptoms or laboratory monitoring or imaging follow-up.^[8–10]^

The exact cause of the bleed into the liver is still unclear. One cause could be accidental biliary tree vascular injury from the guide wire used in the procedure leading to hemorrhage.^[11]^ Another theory suggests that during biliary clearance using a balloon, tractional pressures develop leading to rupture of small biliary blood vessels, resulting in intraparenchymal hemorrhage and subcapsular collection due to centrifugal flow of blood.^[7]^

In our case, HSH was diagnosed 1 day after ERCP but it may take up to 10 days to be diagnosed.^[12]^ The diagnosis should be suspected when patients who underwent an ERCP develop sharp right upper abdominal pain with a drop in hemoglobin levels as well as hypotension and tachycardia. HSH is confirmed by imaging modalities such as abdominal US or CT scan.^[10,13–15]^

Treatment is mainly conservative for persistently stable patients, with prophylactic antibiotics recommended given the risk of infected hematoma.^[16,17]^ Unstable patients with ruptured HSH or peritonitis should undergo surgical treatment. This consists of drainage of the hematoma with hemostasis and serial imaging monitoring. Arterial embolization of the involved vessels can also be taken into account.^[18]^

In our patient, surgery was deemed as too risky due to her labile coagulation blood tests. Arterial embolization was also unavailable in our radiology department.

## 4. Conclusion

HSH is a rare but potentially fatal complication of ERCP. It should be suspected in symptomatic patients who underwent this procedure for diagnostic or therapeutic purposes. The outcomes are generally good, provided diagnosis is made early and the complications are treated accordingly.

## Author contributions

**Conceptualization:** Georgio El Koubayati, Tatiana Charbel.

**Writing—original draft:** Georgio El Koubayati, Tatiana Charbel.

**Writing—review & editing:** Georgio El Koubayati, Randa Choueiry.

**Validation:** Antoine Aoun, Randa Choueiry.

**Visualization:** Antoine Aoun.

**Supervision:** Randa Choueiry.
